# Green synthesis of zinc oxide nanoparticles using Sea Lavender (*Limonium pruinosum* L. Chaz.) extract: characterization, evaluation of anti-skin cancer, antimicrobial and antioxidant potentials

**DOI:** 10.1038/s41598-022-24805-2

**Published:** 2022-11-27

**Authors:** Bassant Naiel, Manal Fawzy, Marwa Waseem A. Halmy, Alaa El Din Mahmoud

**Affiliations:** 1 Environmental Sciences Department, Faculty of Science, Alexandria University, Alexandria, 21511 Egypt; 2 Green Technology Group, Faculty of Science, Alexandria University, Alexandria, 21511 Egypt; 3 National Biotechnology Network of Expertise (NBNE), Academy of Scientific Research and Technology (ASRT), Cairo, Egypt

**Keywords:** Nanoscale materials, Secondary metabolism, Nanobiotechnology, Nanoscale materials, Ecosystem services, Cancer, Biosynthesis, Green chemistry

## Abstract

In the present study, a green, sustainable, simple and low-cost method was adopted for the synthesis of ZnO NPs, for the first time, using the aqueous extract of sea lavender, *Limonium pruinosum* (L.) Chaz., as a reducing, capping, and stabilizing agent. The obtained ZnO NPs were characterized using ultraviolet–visible spectroscopy (UV–VIS), Fourier transform infrared spectroscopy (FT-IR), scanning electron microscopy (SEM), energy-dispersive X-ray analysis (EDX), transmission electron microscopy (TEM), X-ray diffraction (XRD) and thermogravimetric analysis (TGA). The UV–Vis spectra of the green synthesized ZnO NPs showed a strong absorption peak at about 370 nm. Both electron microscopy and XRD confirmed the hexagonal/cubic crystalline structure of ZnO NPs with an average size ~ 41 nm. It is worth noting that the cytotoxic effect of the ZnO NPs on the investigated cancer cells is dose-dependent. The IC_50_ of skin cancer was obtained at 409.7 µg/ml ZnO NPs. Also, the phyto-synthesized nanoparticles exhibited potent antibacterial and antifungal activity particularly against Gram negative bacteria *Escherichia coli* (ATCC 8739) and the pathogenic fungus *Candida albicans* (ATCC 10221). Furthermore, they showed considerable antioxidant potential. Thus, making them a promising biocompatible candidate for pharmacological and therapeutic applications.

## Introduction

With the increasing demand for waste minimization and achieving sustainable development goals through the adoption of the fundamental principles of green chemistry, there is an obvious need for alternative green methods for nanoparticles synthesis^1,2^.

The adoption of green processes in different technologies has been increasingly widespread and is becoming necessary as a result of global environmental problems associated with harsh conventional chemical and physical processes^3–6^.

Nanotechnology has drawn more attention for its cutting-edge nature and wide application range in almost every field of science and technology including biomedical sciences^7,8^. Generally, nanoparticles (NPs) are manufactured using several chemical and physical methodologies, which are quite expensive and pose risks to the environment and human health^6,9–11^.

Plants are the most preferred green and facile route for the synthesis of nanoparticles as they promote large-scale production of stable nanoparticles of various shapes and sizes. Using natural plant extracts is an eco-friendly, simple and cost-effective approach^12,13^. Plant extracts may contain phytochemical compounds such as phenols, flavonoids, alkaloids, terpenes, saponins, and tannins that play as both reducing agents and capping or stabilization agents^14–16^.

Among nanometal oxides, ZnO NPs are known for their antimicrobial^17^, anti-inflammatory and anticancer activity^12,18,19^. They are also used in sunscreens due to their characteristic ultraviolet ray scattering properties^20,21^, drug delivery^22,23^, and wound-healing applications^24,25^. ZnO NPs have several properties as biocompatible multifunctional nanomaterial. The U.S. Food and Drug Administration (FDA) identifies ZnO as a so-called GRAS (= generally recognized as safe) substance^26^. Several studies have demonstrated approaches for the green synthesis of zinc oxide nanoparticles using different plant extracts^17,19,27,28^. However, the sea lavender plant, *Limonium pruinosum* (L.) Chaz., has not been reportedly used for ZnO NPs Synthesis. *Limonium pruinosum* is a halophytic plant that belongs to family Plumbaginaceae. It is a non-succulent, salt excretive, subshrub which lives in two different habitats, coastal salt marsh and desert limestone cliffs^29^.

In the last decade, some plant species has been investigated for their role in the synthesis of nanoparticles^30–33^. However, halophytic and salt marsh dwelling plants have received little attention regarding their potentials for the synthesis of nanoparticles.

Globally, salt marshes provide key ecosystem services contributing several economic, social and ecological aspects^34–36^. Salt marshes are among the most significant blue carbon ecosystems, having a vital role in carbon sequestration, lowering greenhouse gases, and mitigating climate change impacts. Unfortunately, they are vulnerable to severe degradation^37^. Highlighting the resources and potential uses of the species in salt marshes can contribute to the conservation and restoration efforts of these valuable and undermined ecosystem. Therefore, the main objectives of this study are to assess the potentiality of a native salt marsh and common ornamental plant in the Mediterranean basin, *Limonium pruinosum*, for the phytofabrication of ZnO NPs and the possible use of its aqueous extract and synthesized ZnO NPs in biomedical applications.

## Material and methods

### Collection and preparation of plant specimens

Plant specimens (Supplementary Fig. 1) were collected from their natural habitats, the salt marshes along the northwestern Mediterranean coast of Egypt, particularly from El-Alamein (Latitude 30° 55′ 338″, Longitude 28° 29′ 365″, Altitude 11). Plant material was collected in accordance with applicable national and international guidelines^38^. Permission for collecting the investigated plant species for scientific purposes was obtained from Environmental Sciences Department, Alexandria University. Plant specimens were identified by Dr. Marwa Waseem A. Halmy according to Boulos^39^. Voucher specimens were deposited in Tanta University Herbarium (TANE) with voucher Numbers: 14200–14210, which is a public herbarium providing access to the deposited material. The shoot system, leaves and stems, were utilized in this investigation. Specimens were carefully washed using tap water then distilled water, to remove soil particles. Afterword, the specimens were dried at 60 °C to constant weight then grounded to fine powder using a stainless-steel grinder Moulinex 700 W, France. Plant powder was preserved in airtight jars for subsequent use.

#### Preparation of plant extract

Two grams of plant powder were stirred and heated at 70 °C in 100 ml distilled water for 30 min. The extract was allowed to cool to room temperature. The pH of plant extract was 7.84. The extract was filtered using Whatman filter paper No.1 and stored at 4 °C for subsequent experimental use.

#### Green synthesis of ZnO NPs

Zinc acetate dihydrate {Zn (CH_3_COO)_2_·2H_2_O} and Sodium Hydroxide (NaOH) were purchased from Loba Chemie, India.

Briefly, 2.5 ml of plant extract was added to 25 ml 0.5 M Zinc acetate dihydrate. The pH of the mixture was 6.13, 2 M NaOH was added drop wise to maintain pH at 8, then the mixture was then stirred and heated at 70 °C for 30 min for complete reduction and formation of a white precipitate.

**Figure 1:**
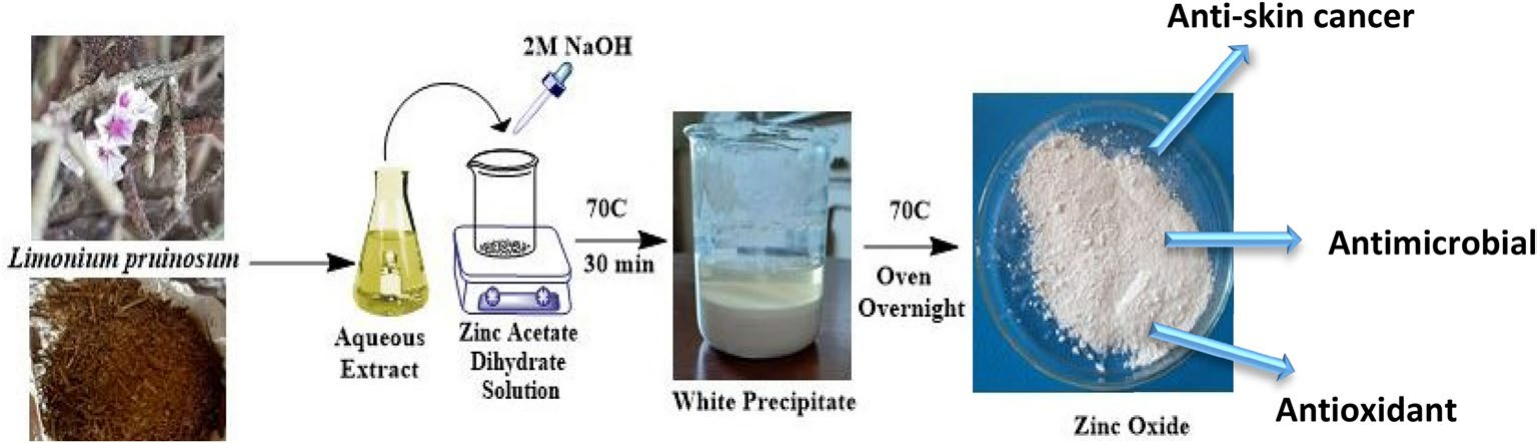
Schematic illustration of the phytosynthesis of ZnO NPs.

The resulting material was then collected via decantation, washed with distilled water to remove residuals and oven dried at 70 °C for overnight to yield powdered ZnO nanoparticles^17,40,41^. The synthesis steps are demonstrated in Fig. 1. The dried sample was stored at room temperature in airtight container for further characterization and applications. The yield percentage was then estimated according to the following formula:

1 $$\mathrm{Yield }(\mathrm{\%}) = (\mathrm{Experimental\, weight\, of\, ZnO}/\mathrm{Theoretical\, weight \,of \,ZnO}) \times 100$$

### Characterization of ZnO NPs

The synthesized ZnO NPs from the aqueous extract of *L. pruinosum* were confirmed and characterized using double-beam UV–visible spectrophotometer (T70/T80 series UV/Vis Spectrophotometer, PG Instruments Ltd, U.K.) in the range of 200–800 nm to observe the characteristic peak confirming ZnO NPs formation.

Fourier transform infrared spectroscopy (FT-IR) (Nicolet iS50 FTIR Spectrometer, Thermo Fischer Scientific, Japan) in the range of 4000–400 cm^−1^ and gas chromatography-mass spectrometry (GC–MS) (Trace GC1310-ISQ mass spectrometer, Thermo Scientific, Austin, TX, USA) were both used for the determination of the functional groups and phytoconstituents contributing to the reduction and stabilization of the ZnO NPs. Scanning electron microscope, energy dispersive X-ray analysis (EDX) using (JEOL, JSM IT 200, Japan), transmission electron microscope using (JEOL, JSM-1400 PLUS, Japan) and X-ray diffractometer (Bruker D8 Discover Diffractometer, USA) were used to analyze the surface morphology, identify the elemental composition, size, and shape of green synthesized ZnO NPs. Moreover, thermogravimetric analysis was conducted using Labsys evo Setaram, France, for the determination of the thermal stability of the green synthesized ZnO NPs.

### Anti-skin cancer/cytotoxicity

Evaluation of cell viability for green synthesized ZnO NPs was performed using MTT assay against A-431(Skin cancer/Epidermoid Carcinoma) and WI-38 (Normal lung fibroblast cells); purchased from Vacsera center, Giza, Egypt; at different concentrations of ZnO NPs and *L. pruinosum* extract (31.25, 62.5, 125, 250, 500 and 1000 µg/ml). Methylthiazolyl diphenyl-tetrazolium bromide (MTT) assay is the most commonly used method for assessing metabolic activity of cells. It is a reliable colorimetric and quantitative assay based on the ability of the cellular mitochondrial dehydrogenase enzyme to cleave the yellow water soluble MTT to produce insoluble dark blue/purple formazan deposits in living cells used^42^.

Briefly, a 96-wells tissue culture plate was inoculated with 1 ×10^5^ cells/ml (100 µl/well) and incubated at 37 °C for 24 h. Growth medium was then decanted. Two-fold dilutions of the tested sample were preserved in RPMI medium with 2% serum. The wells were treated using 0.1 ml of each dilution and 3 wells were used as control, receiving only serum. The plate was incubated at 37 °C and then examined. Morphological changes of cells were investigated. MTT solution was prepared (5 mg/ml in PBS) (BIO BASIC CANADA INC). About 8- 20 µl of MTT solution were added to each well, thoroughly shaken for 5 min, and incubated (37 °C, 5% CO_2_) for 4 h till formation of formazan. After that, formazan was resuspended in 200 µl DMSO and shaken thoroughly for 5 min. Absorbance was measured at 560 nm^43–45^. Experiment was performed in triplicate. The concentration of ZnO NPs and *L. pruinosum* extract required to inhibit the growth of the skin cancer cells (A-431) by half was calculated from the dose–response curve and represented as IC_50_.

### Antimicrobial activity

The antimicrobial activity of green synthesized ZnO NPs, *L. pruinosum* extract, and Gentamycin was tested against 6 different pathogenic microorganisms including Gram positive bacteria (*Bacillus Subtilis* (ATCC 6633), *Staphylococcus aureus* (ATCC 6538), Gram negative bacteria (*Escherichia coli* (ATCC 8739), *Enterobacter aeruginosa*) and pathogenic fungi (*Candida albicans* (ATCC 10221) and *Aspergillus flavus*).

Antimicrobial activity was determined by using the agar well diffusion method^46,47^. Ten mg/ml of all samples were dissolved in normal saline (0.9% NaCl). Saline did not have antimicrobial activity against all tested pathogenic strains. Gentamycin was tested as a positive control. One hundred µl of green synthesized ZnO NPs, *L. pruinosum* extract, and Gentamycin was tested.

### Antioxidant activity

Antioxidant activity of green synthesized ZnO NPs and *L. pruinosum* extract were determined using 2,2-diphenyl-1-picrylhydrazyl (DPPH) assay. In brief, 1 ml of 0.1 Mm DPPH solution was added to 3 ml of green synthesized ZnO NPs and plant extract in ethanol at different concentrations (1.95, 3.9, 7.8125, 15.625, 31.25, 62.5, 125, 250, 500, 1000 µg/ml). These concentrations were prepared by dilution method. Ascorbic acid was used as standard. The control DPPH was measured without sample. The mixture was shaken vigorously and allowed to stand at room temperature for 30 min. In order to measure the absorbance a spectrophotometer (UV–VIS Milton Roy, USA) was used at 517 nm. The assay was conducted in triplicate. The IC_50_ value was calculated using Log dose inhibition curve. The percentage of DPPH scavenging effect was calculated using the following equation:

DPPH scavenging effect (%)^16,30^:

2 $${\text{A}}_{0} - {\text{A}}_{{1}} /{\text{A}}_{0} \times {1}00$$

where A_0_ was the absorbance of the control reaction and A_1_ was the absorbance in the presence of test.

## Results and discussion

### Characterization of ZnO NPs

After the addition of plant extract to Zinc acetate dihydrate precursor and NaOH, white precipitate was formed indicating the phytofabrication of ZnO NPs with a yield equal to 88.2%. This finding supports large scale production of ZnO NPs using plant-mediated synthesis procedure close to chemical methods^48^.

#### UV–Vis spectra analysis

**Figure 2:**
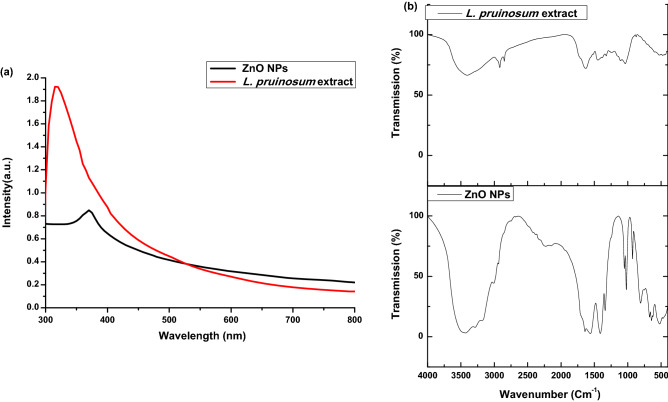
(**a**) UV–Vis spectrum; (**b**) FT-IR of *L. pruinosum* extract and the phytosynthesized ZnO NPs.

The obtained ZnO NPs were suspended in deionized water and sonicated for 10 min to detect the UV–visible spectra. The UV–Vis spectra showed a strong absorption peak at about 370 nm confirming the successful formation of ZnO NPs using aqueous extract of *L. pruinosum*. Moreover, the UV–Vis spectra of plant extract showed absorption peak at 320 nm indicating the presence phenolic compounds as illustrated in Fig. 2a. This result is in agreement with other previous studies^49–51^.

#### Fourier transform infrared spectroscopy (FT-IR)

The FT-IR revealed the presence of a characteristic peak at about 523 cm^−1^ which indicated the formation of Zn˗O nanostructure stretching similarly^52^. The vibrational peaks observed at around 3433 and 3288 cm^−1^ could be corresponding to hydroxyl (OH) groups which are observed at around 3407 of *L. pruinosum* and are possibly related to phenolic and alcoholic compounds*.* However, the peak projected around 2937 cm^−1^ corresponds to the C-H stretching. The peak at around 1050 cm^−1^ is corresponding to the stretching of the –CN group which was observed at around 1035 cm^−1^ of *L. pruinosum*.

In addition, the vibrational peaks at 1640 cm^−1^ and 1560 cm^−1^ were corresponding to the H–O–H bending as shown in Fig. 2b. Similarly, the H–O–H bending was observed at around 1630 cm^−1^ of *L. pruinosum*. The peaks mentioned above confirm the presence of phytochemicals such as terpenoids and phenolics in the plant extract that were involved in the reduction and stabilization of ZnO NPs. These results are supported by other previous findings^18,53,54^. Moreover, plant extract is also further characterized using GC–MS to define the major compounds involved in the synthesis process.

#### Phytochemical screening

The phytochemical analysis of plant extract was carried out using GC–MS to determine phytochemicals that may be involved in the reduction and stabilization of ZnO NPs as presented in Supplementary Fig. 2 and Table 1. The identification of phytochemicals was conducted using WILEY 09 and NIST 11 mass spectral databases based on a comparison of their retention times and mass spectra^55,56^.

**Table 1:** Bioactive compounds identified in aerial parts of *L. pruinosum* extract.

No.	Compound name	Chemical structure	Category	Retention time (min)	Area (%)
1	Carvone	C_10_H_14_O	p-Menthane Monoterpenoid	12.79	2.93
2	Bergamotol, Z-à-trans-	C_15_H_24_O	Sesquiterpenoid	19.72	6.13
3	6-epi-shyobunol	C_15_H_26_O	Sesquiterpenoid	23.09	11.51
4	Bisabolol oxide A	C_15_H_26_O_2_	Oxanes	25.10	6.49
5	Hexadecanoic acid, TMS	C_19_H_40_O_2_Si	Ester	31.29	11.67
6	Oleic Acid, (Z)-, TMS derivative	C_21_H_42_O_2_Si	Ester	34.37	3.66
7	Monolinolein, TMS	C_27_H_54_O_4_Si_2_	Ester	37.93	2.16

Totally 7 major compounds were identified from GC–MS chromatogram belonging to different categories including p-menthane Monoterpenoid, Sesquiterpenoid, Oxanes, and Esters. The major constituents were Hexadecanoic acid, TMS (11.67%), 6-epi-shyobunol (11.51%), Bisabolol oxide A (6.49%), Bergamotol, Z-à-trans- (6.13%), Oleic Acid, (Z)-, TMS derivative (3.66%), Carvone (2.93%) and Monolinolein, TMS (2.16%) in descending order of percentage. These phytochemicals may be incorporated in the phyto-reduction of nanoparticles^57,58^.

Based on UV–Vis, FT-IR and GC–MS results it can be concluded that *L. pruinosum* extract consists of several phytochemicals including; alcohols, phenols, terpenoids and esters which acted as reducing and stabilizing agent for successful phytosynthesis of ZnO NPs.

#### Morphological structure

**Figure 3:**
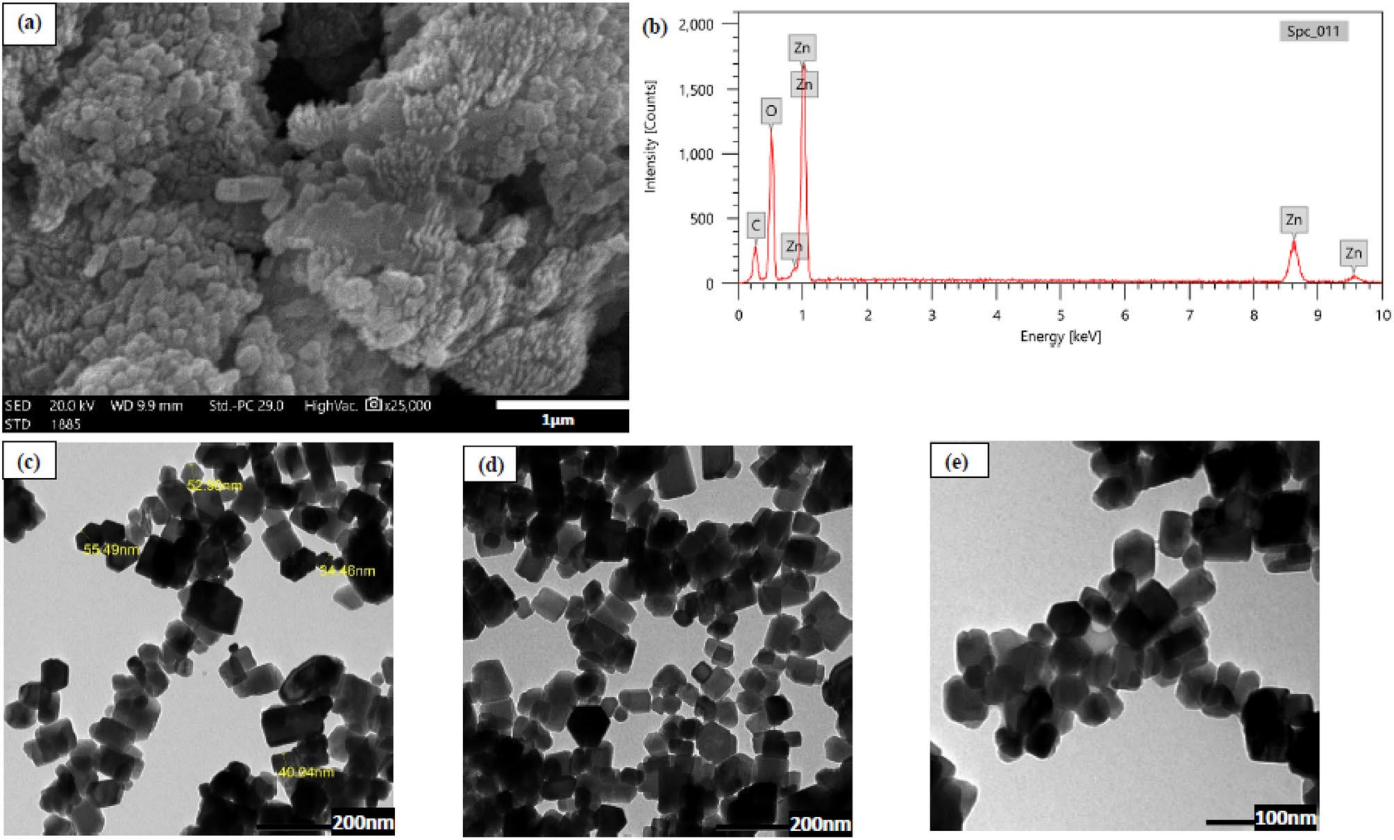
(**a**) SEM; (**b**) EDX; (**c**–**e**) TEM images of the phytosynthesized ZnO NPs.

Scanning electron microscope (SEM), energy dispersive X-ray analysis (EDX), and transmission electron microscope (TEM) were used to analyze the surface morphology, size, and shape of green synthesized ZnO NPs as illustrated in Fig. 3.

Figure 3 a shows that the obtained ZnO NPs are crystalline in nature. The EDX results confirmed the purity of the obtained ZnO NPs and the presence of Zinc in its oxide form as shown in Fig. 3b. Strong emission peaks of Zn were detected at ~ 1 keV, 8.6 keV and 9.6 keV. The detected emission peaks of carbon at ~ 0.3 keV and oxygen at ~ 0.5 keV might be due to the plant biomass used in phyto-synthesis. These findings are consistent with that of Barzinjy and Azeez^59^. The TEM images showed the hexagonal/cubic shape of green synthesized ZnO NPs with an average size of ~ 41 nm as shown in Fig. 3c–e. Several studies reported various shapes and size of the biosynthesized ZnO NPs as illustrated in Table 2.

**Table 2:** Comparison of biosynthesized ZnO NPs using different plant species.

Plant species	Shape	Size (nm)	References
*Artocarpus gomezianus* Wall. ex Trécul	Spherical	10–30	^60^
*Berberis aristata* DC	Needle	20–40	^61^
*Artocarpus heterophyllus* Lam	Spherical	12–24	^62^
*Prosopis farcta* (Banks & Sol.) J.F. Macbr	Hexagonal	40–50	^63^
*Phoenix dactylifera* L	Spherical	22	^64^
*Avena Sativa* L	Hexagonal	100	^65^
*Limonium pruinosum* (L.) Chaz	Hexagonal/cubic	~ 41	Present study

#### X-ray diffraction (XRD)

**Figure 4:**
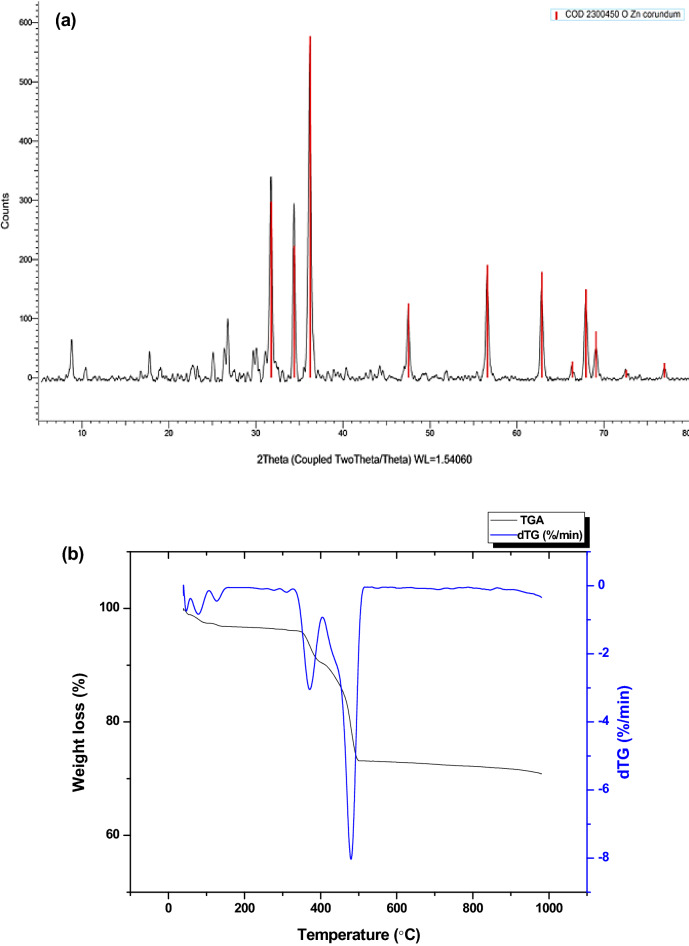
(**a**) XRD pattern and (**b**) TGA pattern of the phytosynthesized ZnO NPs.

Dried ZnO NPs were used for XRD analysis with Cu-Kɑ radiation (λ = 1.54060 A°), the relative intensity data were collected over a 2Ө range of 5–100 degrees. The XRD pattern of ZnO NPs (Fig. 4a) reveals sharp peaks that indicate the purity and crystallinity of green synthesized ZnO NPs. The 2Ө angles of the diffraction peaks were located at ~ 31.45°, 34.66°, 36.26°, 47.48°, 56.29°, 62.7°, and 68.31°. These peaks are similar to those reported in other studies^47,59^.

The peaks were paralleled to JCPDS Card (2300450) confirming the hexagonal phase of ZnO NPs with space group P 63 mc (186). XRD analysis detected the average crystalline size of obtained ZnO NPs ~ 11 nm using Debye–Scherrer’s equation^66^.

Size difference between XRD and above-mentioned TEM was possibly attributed to the fact that XRD gives information about the grain size and larger particles may be polycrystalline as a result of the coalescence of smaller grains^67,68^.

#### Thermogravimetric analysis (TGA)

The thermogravimetric analysis was conducted using (Labsys evo Setaram, France) for the determination of thermal stability of green synthesized ZnO NPs as shown in Fig. 4b. The weight loss of the ZnO NPs dried powder was measured while subjected to thermal fluctuations from room temperature to 1000 °C. The heating was carried out under Nitrogen atmosphere with temperature scanning rate 10 °C/min.

It was shown that the green synthesized ZnO NPs exhibited a high thermal stability. As a gradual weight loss up to about 29.08% of actual weight at the temperature range from room temperature to 1000 °C, which is likely due to loss of moisture content and organic substances in the samples^27,33,54^.

In contrast, Barzinjy and Azeez^59^ observed a considerable weight-loss (~ 65%) between 350 and 600 °C, which was attributed to the degradation of organic groups involved in the biosynthesis of ZnO NPs using plant extract. These results indicated that the plant-mediated synthesis of ZnO NPs was thermally stable.

### Anti-skin cancer/cytotoxicity

**Figure 5:**
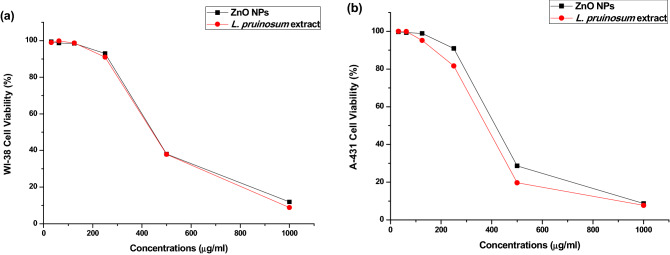
Cell viability % efficiency of the phytosynthesized ZnO NPs and *L. pruinosum* extract against (**a**) normal lung fibroblast cells (WI-38) and (**b**) cancerous cells (A-431).

The MTT assay results demonstrated that cancer cell viability decreased significantly to 28.6% at 500 µg/ml of ZnO NPs after 24 h exposure. The anticancer activity of ZnO NPs exhibited dose-dependent profile as shown in Fig. 5 in agreement with previous studies^69,70^.

The outcomes were in accordance with that of the study conducted by Nilavukkarasi et al.^12^ that examined lung cancer cell lines (A549) using cell proliferation assay at different concentrations (1.45, 3.9,7.8, 15.6, 31.2, 62.5, 125, 250, and 500 µg/ml) of synthesized ZnO. Major alterations of cells were observed at higher concentrations such as cell shrinkage and extensive cell detachment, which is consistent with the outcomes of the current study as shown in Supplementary Fig. 3a,b.

The concentration of ZnO NPs required to inhibit the growth of the skin cancer cells (A-431) by half calculated from the dose–response curve IC_50_ = 409.7 μg/ml. Lingaraju et al.^71^ have reported that the (IC_50_) was found to be 383.05 μg/ml and 329.67 μg/ml for lung (A549) and Hepatocarcinoma (HePG2) cell lines, respectively.

The green synthesized ZnO NPs in the current study showed higher toxicity towards cancer cells compared to normal cells (WI-38), IC_50_ of ZnO NPs was 568.59 and 409.7 µg/ml against normal cells and cancerous cells, respectively.

Based on previous studies, green synthesized ZnO NPs showed high biocompatibility and less toxicity towards normal cells^7^. For instance, the green synthesized ZnO NPs using *Crotalaria verrucosa* leaf extract was tested against HeLa and DU145 cell lines and cytotoxicity assay revealed its selectivity for cancer cells highlighting its potential as substitute therapy against cervical and prostate cancer^72^.

The possible mechanism of anticancer effect of ZnO NPs may be attributed to oxidative stress and apoptosis^62,73^.

Anticancer activity of *L. pruinosum* extract was also examined and the results demonstrated that plant extract showed higher activity than green synthesized ZnO NPs, the IC_50_ of plant extract was 554.17 and 362.74 µg/ml against normal cells and cancerous cells respectively as shown in Fig. 5a,b and confirmed by microscopic examination as shown in Supplementary Fig. 3a,b. This may be attributed to phytochemical content of *L. pruinosum* species particularly Hexadecanoic acid, as it has been known that these compounds may have the ability to halt cancer cell growth^74–77^. Several previous studies also confirmed the potential anticancer activity of green synthesized ZnO NPs and plant phytochemicals^63,78,79^. Furthermore, based on our investigation, both *L. pruinosum* extract and its green synthesized ZnO NPs showed no cytotoxic effect on normal cells (WI-38) up to 250 µg/ml compared to studies performed by Jobie et al.^80^ and Chen et al.^81^ who observed that ZnO NPs are toxic to normal vero cells at concentrations of 120 and 100 µg/ml, respectively. These findings suggest the high biocompatibility of *L. pruinosum* extract and ZnO NPs.

### Antimicrobial activity

The results of antimicrobial activity of green synthesized ZnO NPs, *L. pruinosum* extract and Gentamycin tested against six different pathogenic microorganisms are shown in Table 3.

**Table 3:** Inhibition zones (diameter in mm) of phytosynthesized ZnO-NPs and *L. pruinosum* extract against different pathogenic microorganisms.

Pathogenic microorganism	Limonium	ZnO NPs	Control (gentamicin)
*Bacillus subtilis* (ATCC 6633)	20	24	22
*Staphylococcus aureus* (ATCC 6538)	24	26	15
*Escherichia coli* (ATCC 8739)	31	29	17
*Enterobacter aeruginosa*	16	20	16
*Candida albicans* (ATCC 10221)	29	28	23
*Aspergillus flavus*	11	14	20

The results revealed that the synthesized ZnO NPs and plant extract showed the highest inhibition zone versus *E. coli* with 29 and 31 mm followed by *C. albicans* with 28 and 29 mm, respectively. Plant extract and its green synthesized ZnO NPs exhibited strong antimicrobial activity over conventional antibiotic Gentamycin except for *A. flavus.* It has been previously reported that ZnO NPs have significant antimicrobial activity^40,79,82,83^. Antimicrobial activities of halophytes were attributed to phenolics due to their hydroxyl functional groups, degree of polymerization and lipophilicity; enhancing their binding to bacterial cell membrane as extensively discussed studies^84,85^. Guimarães et al.^86^ also investigated the antibacterial mechanism of action of phytochemicals and it was attributed to disruption of cellular membrane and eventually cell death. The mechanism of action of ZnO NPs depends on binding and interacting to the cell membrane and accumulation in the lipid layer leading to inhibition of enzymes, DNA and ATP synthesis promoting cell lysis^17^. Moreover, Soltanian et al.^41^ and Sirelkhatim et al.^87^ demonstrated that ZnO NPs induce excessive reactive oxygen species generation and cell wall damage.

### Antioxidant activity

**Figure 6:**
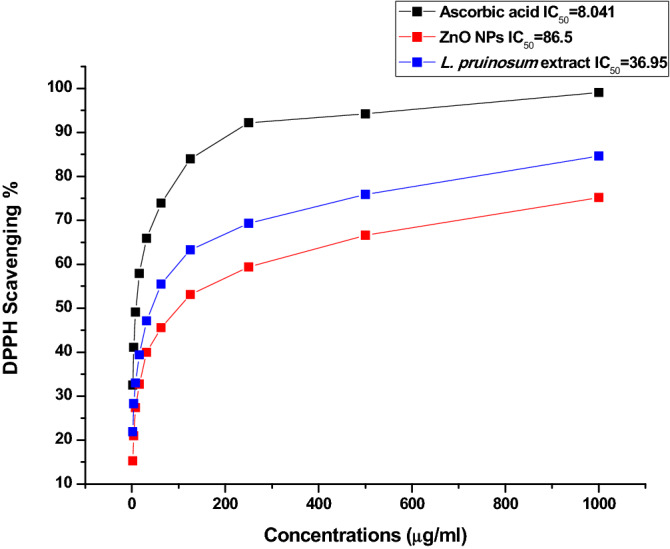
Antioxidant activity of ZnO NPs, *L. pruinosum* extract and Ascorbic acid.

The results presented in Fig. 6 revealed that ZnO NPs exhibited considerable antioxidant activity via the scavenging of free radicals^88^ in a dose-dependent manner^89^. The highest DPPH activity was recorded at 1000 μg/ml as 75.2% for ZnO NPs and 84.6% for *L. pruinosum* extract. In the present study the IC_50_ of ZnO = 86.5 μg/ml, whereas Loganathan et al.^15^ have reported IC_50_ = 95.80 μg/ml. *L. pruinosum* extract showed relatively higher antioxidant activity than their phyto-synthesized ZnO NPs due to the presence of secondary metabolites as confirmed by FT-IR and GC–MS results. For instance, phenolics and terpenoids are potent antioxidants. These phytochemicals also acted as capping agent for ZnO NPs and possibly related to their antioxidant activity^90^. Moreover, Lopes et al.^84^ and Bose et al.^91^ demonstrated that halophytes have high antioxidant capability to tolerate salinity stress and rich in phenolic content with remarkable antioxidant activity.

## Conclusion

The current study demonstrated the efficiency of the aqueous extract of sea lavender, *Limonium pruinosum*, as reducing, capping and stabilizing agent leading to the successful synthesis of hexagonal/cubic zinc oxide nanoparticles. To the best of our knowledge, this study is the first to report the green synthesis of ZnO NPs using the aqueous extract of this halophytic plant species and the use of zinc acetate dihydrate as a precursor. The green synthesized ZnO NPs exhibited considerable anticancer activity against A-431 cell lines. Both plant extract and its green synthesized ZnO NPs showed no cytotoxic or detrimental effect on normal cells (WI-38) up to 250 µg/ml supporting its biocompatibility. Also, ZnO NPs along with plant extract exhibited remarkable antimicrobial activity compared to Gentamycin. Moreover, they showed comparable antioxidant activity. These findings suggest the adoption of *Limonium pruinosum* ZnO NPs as cost effective and ecofriendly candidate for biomedical and therapeutic applications. The present study adds more support for the urgent need for the conservation and sustainable management of salt marshes ecosystems and its halophytic plant species not only for their undeniable role in carbon sequestration but also as a sustainable resource for green synthesis of nanomaterials.

## Supplementary Information

Supplementary Information.

## Data Availability

All data generated or analyzed during this study are included within the article.
