# Supplementary material for: Green synthesis of zinc oxide nanoparticles using Sea Lavender (Limonium pruinosum L. Chaz.) extract: characterization, evaluation of anti-skin cancer, antimicrobial and antioxidant potentials
Source: Sci Rep. 2022 Nov 27;12:20370. doi: 10.1038/s41598-022-24805-2 (PMC9701696; doi:10.1038/s41598-022-24805-2)
Supplement: Supplementary file 1 — Supplementary Information. [file 41598_2022_24805_MOESM1_ESM.pdf]

Supplementary Material

Green synthesis of zinc oxide nanoparticles using Sea Lavender *(Limonium pruinosum* L. Chaz.) extract: Characterization, evaluation of anti-skin cancer, antimicrobial and antioxidant potentials.

Bassant Naiel[1*], Manal Fawzy[1,2,3], Marwa Waseem A. Halmy[1], Alaa El Din Mahmoud[1,2]

[1]Environmental Sciences Department, Faculty of Science, Alexandria University, 21511, Alexandria, Egypt.

[2]Green Technology Group, Faculty of Science, Alexandria University, 21511, Alexandria, Egypt.

[3]National Egyptian Biotechnology Experts Network, National Egyptian Academy for Scientific Research and Technology, Egypt.

*Corresponding author: Bassant Naiel; bassant_hassan22@yahoo.com

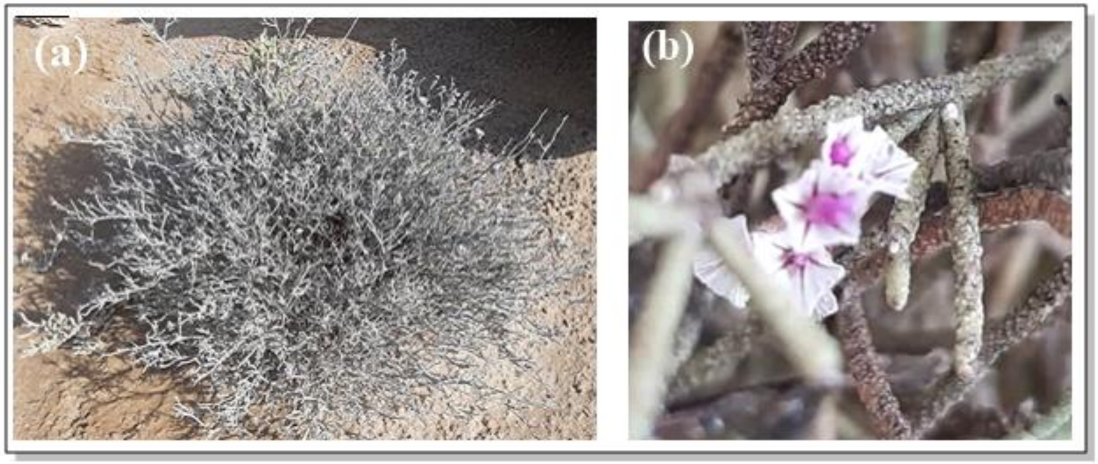

Supplementary Figure 1. *L. pruinosum* (L.) Chaz, (a): life form; and (b): flowers.

Limonium pruinosum

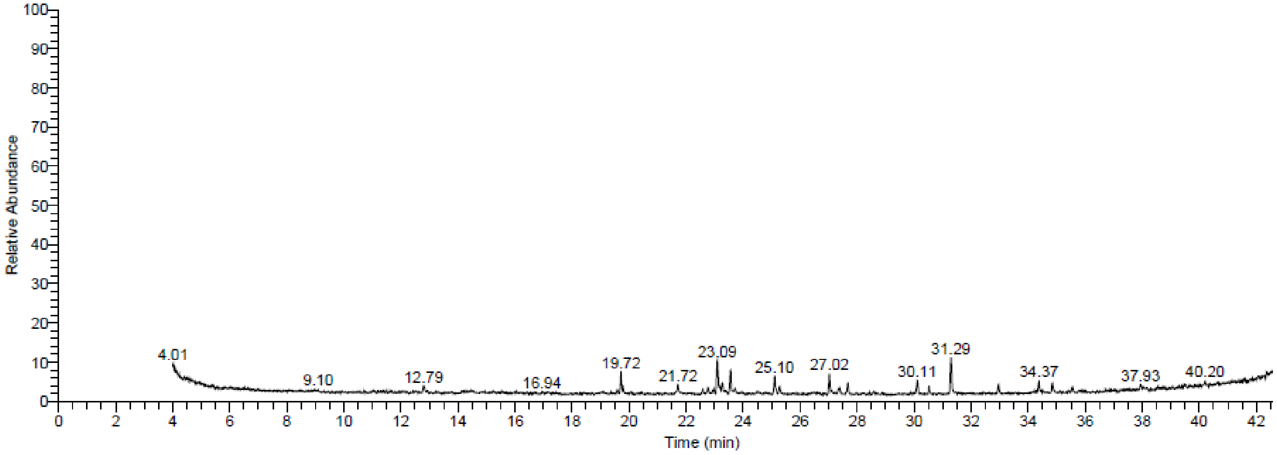

Supplementary Figure 2. GC-MS analysis of *L. pruinosum* extract

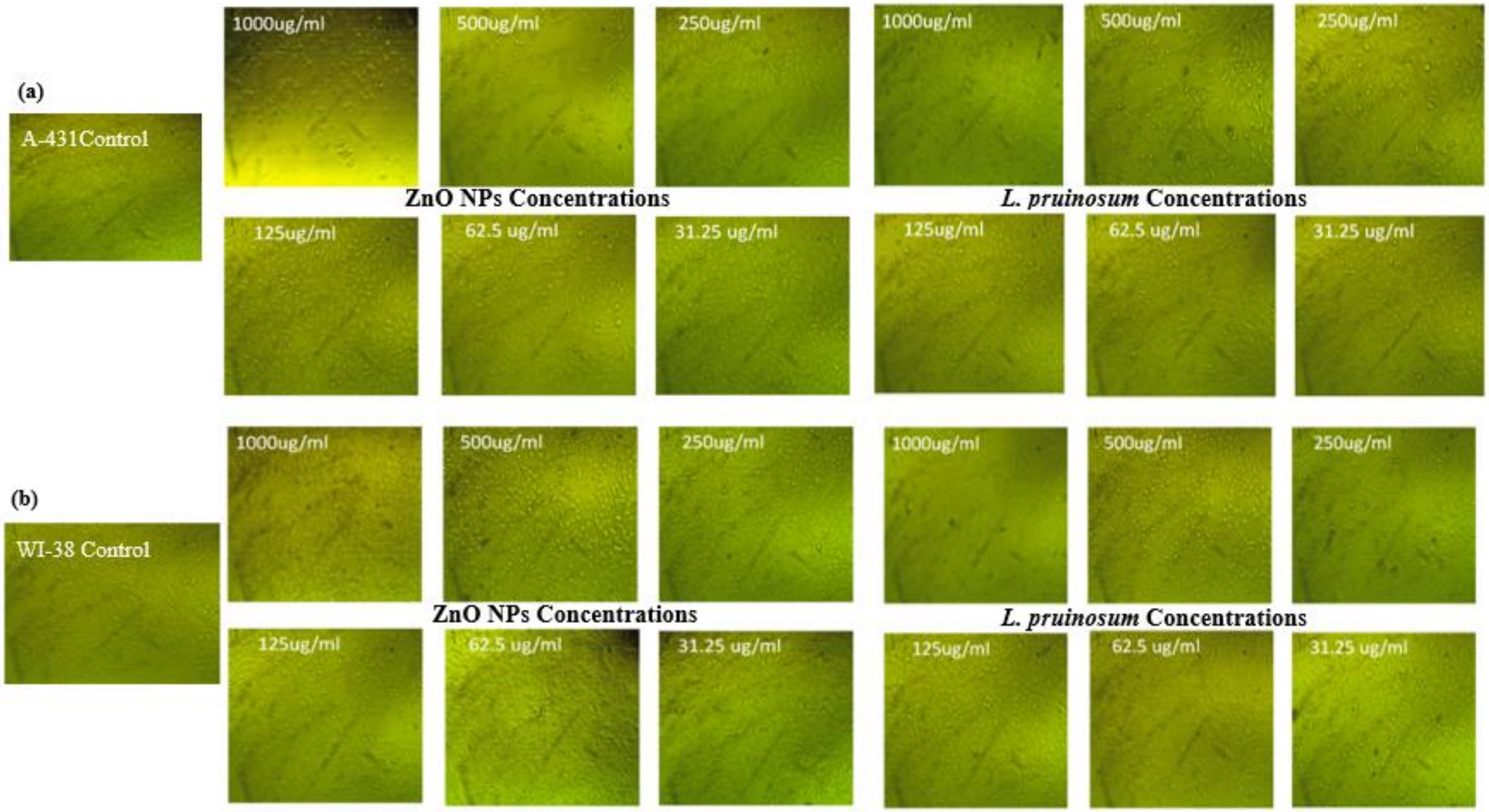

Supplementary Figure 3. Morphological changes of (a): A-431 cancerous cells and (b): WI-38 normal cells at different concentrations of ZnO NPs & *L. pruinosum* extract
